# Simulation of the electrolyte imbalance in vanadium redox flow batteries

**DOI:** 10.1371/journal.pone.0318460

**Published:** 2025-02-07

**Authors:** Baowen Zhang, Yuan Lei

**Affiliations:** 1 Aviation Engineering School, Air Force Engineering University, Xi’an, P. R. China; 2 Equipment Management and Unmanned Aerial Vehicle Engineering School, Air Force Engineering University, Xi’an, P. R. China; 3 School of Chemical Engineering, Northwest University, Xi’an, China; Washington State University, UNITED STATES OF AMERICA

## Abstract

The stack is the core component of large-scale flow battery system. Based on the leakage circuit, mass and energy conservation, electrochemicals reaction in porous electrode, and also the effect of electric field on vanadium ion cross permeation in membrane, a model of kilowatt vanadium flow battery stack was established. The electro chemical reaction parameters, ion concentration and temperature of each single cell in the stack were calculated respectively. The imbalance of vanadium ion concentration and the effects of current density and electrolyte temperature on the electrolyte imbalance in the stack were studied.

## 1. Introduction

Energy storage system is an important technology applied to large and medium-sized energy systems, and has great prospects in smoothing renewable energy power output [1–3]. Among all kinds of energy storage systems, all-vanadium flow batteries (VRFBs) have attracted much attention[4–7]. The most significant advantage of VRFBs is the independence of battery capacity and power [8–11]. The battery capacity depends on the total amount of vanadium ions in the electrolyte, and the battery power depends on battery stack structure and operating parameters such as current density [12–14]. The independence of vanadium battery capacity and power is of great significance to the large-scale production and application of the whole battery system. By changing the electrolyte capacity and battery stack structure respectively, vanadium battery system can form different capacity and power combinations to meet various load requirements with great flexibility [15–17]. Though the large-scale all-vanadium flow battery system can operate efficiently and durably through reasonable structure design and operation condition control, its wide-spreading still requires efficient battery stack, stable electrolyte and advanced battery control system [18]. To ensure the steady and efficient operation of vanadium battery, it is very important to control the concentration of positive and negative electrolyte. Vanadium ions, serving as active materials, flow within the electrolyte circulation of the positive electrode and negative electrode respectively, during the charge and discharge process of vanadium battery. However, a small amount of vanadium ions permeates through the membrane and react with vanadium ions on the other side, resulting in the difference in total concentration of vanadium ions between the positive and negative sides. Thus, the capacity of VRFBs decrease due to the imbalance of vanadium ions in electrolyte.

The analysis of material, energy and charge transfer mechanism in vanadium batteries is an important basis for developing effective methods to suppress electrolyte imbalance. Many scholars have done a lot of work in experimental research and model development of VRFB system. Oreiro et al. studied the permeation of vanadium ions across membrane and measured the diffusion coefficient of vanadium ions in various valence states. The concentration change of vanadium ions in the battery stack was analyzed [19]. Zou et al. introduced a dynamic model to predict electrolyte capacity fade in VRFBs. The capacity fade through ion crossover and electrolyte volume change was investigated. The model accurately forecasted electrolyte volume and capacity changes, enhancing VRFB reliability in energy storage [20]. Puleston et al. presented a comprehensive analysis of the impact of electrolyte imbalances on battery capacity. They introduced indicators to measure different types of imbalances and revealed that remixing could be detrimental under certain conditions. The study also showed that inducing an optimal mass imbalance could mitigate capacity loss from oxidation, proposing a systematic procedure to track this optimum [21]. Shah et al. established a series of VRFB models and analyzed the mass and heat transfer processes [22–24]. Xu et al. developed a three-dimensional model of vanadium battery and studied the influence of battery channel structure on battery performance [25]. Tang et al. established a mass and heat transfer model for VRFB system including vanadium ion cross-permeation, in which all parameters of the stack are assumed to be uniform [26,27]. Bureš et al. established a physical-mathematical model coupling mass transfer, heat transfer and electrochemical processes in VRFBs, simulating the voltage and electrolyte changes during charge and discharge of vanadium battery [28]. Wang et al. introduced a novel dynamic model for VRFBs to investigate capacity loss mechanisms. Their work provided insights into capacity loss factors and offered practical mitigation strategies, aiding in developing re-balancing techniques and optimization methods for efficient battery operation with minimal computational load [29]. Nevertheless, more efficient model should be derived to evaluate the electrolyte imbalance in stack.

In this paper, the model of vanadium flow battery was established to describe the transport process in each cell. The imbalance of total electrolyte concentration between anode and cathode of VRFBs, the difference of vanadium ion between different cells in kilowatt stack module and its influencing factors were analyzed.

## 2. Physical and mathematical model of vanadium flow battery system

**Fig 1:**
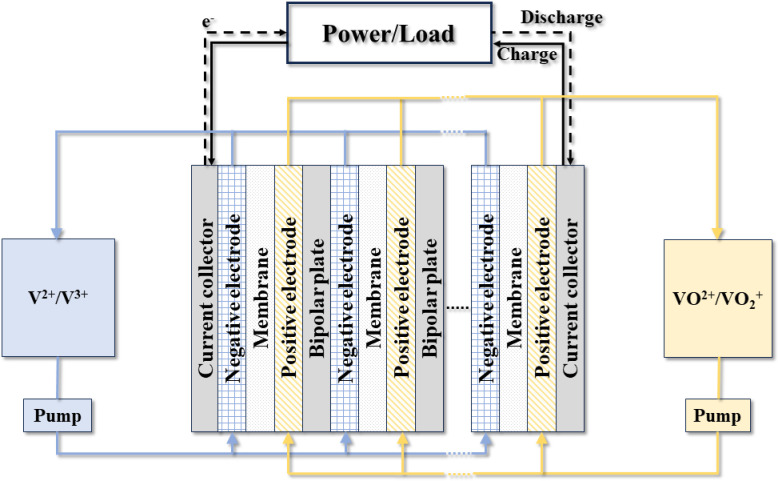
Schematic diagram of VRFBs.

Within each cell of the stack, The ion exchange membrane separates two porous electrodes that are in contact with bipolar plates on either side, as shown in Fig 1. Vanadium ions in electrolyte react electrochemically on surface of porous electrode when electrolyte flows through porous electrode during charge and discharge stages. In the charging process, trivalent vanadium ion (V^3+^) at the cathode gets electrons to generate divalent vanadium ion (V^2+^), and tetravalent vanadium ion (VO^2+^) at the anode loses electrons to generate pentavalent vanadium ion (VO_2_^+^).

A series of simplified assumptions are introduced into the model to simplify the complex processes involved in the system, allowing for more manageable calculations and analysis.

Under high flow rate conditions, mixing may not be perfect, leading to localized concentration variations that could affect the system's performance. However, flow rate in electrolyte of VRFBs usually relatively low. Thus, it is reasonable to assume a uniform distribution of substances in the cell and storage tank, as well as immediate mixing of the incoming electrolyte, facilitates the modeling of concentration gradients and mass transfer processes. The distribution of each vanadium ions in the cell and the storage tank is uniform, and the electrolyte flowing into the storage tank is immediately mixed uniformly.

In the practical applications, the volume of the electrolyte may change due to evaporation or absorption. Moreover, variations in flow rate can also occur due to pump pressure changes in system. These factors could affect the system's efficiency and reliability. However, these factors has very little influence on the vanadium ion concentration in every charge-discharge stage. Maintaining a constant electrolyte volume and flow rate simplifies the energy and mass balance calculations. Thus, the volume of the electrolyte does not change during the charging and discharging process and the electrolyte flow rate in each cell is the same.

The side reactions such as hydrogen evolution and oxygen evolution can consume energy and produce byproducts that could affect the system's performance. For example, hydrogen evolution can lead to the formation of bubbles that can disrupt the flow of electrolyte and reduce the effective surface area for electrochemical reactions. However, the reaction rates of these side reactions are very slow. Ignoring these side reactions simplifies the electrochemical reactions and reduces the complexity of the model. Herein, side reactions such as hydrogen evolution and oxygen evolution are ignored.

In reality, there may be a delay between the permeation of ions and their reaction due to diffusion limitations or surface fouling. However, the concentration of vanadium ions permeating through across the membrane is rather small and assuming that vanadium ions penetrating through the ion exchange membrane react immediately simplifies the transport and reaction processes. Therefore, vanadium ions permeating into the cell through the ion exchange membrane react immediately.

In a word, the assumptions introduced into the model simplify the analysis and calculations with their implications considered, guaranteeing the predictive accuracy of the model sufficiently at the same time. This will help ensure the model accuracy and provide reliable predictions and insights.

According to the Nernst equation, the voltage of each cell is expressed as:


$$E_{cell,n}=E_{0}+\frac{RT}{F}\ln\left(\frac{c_{2,n}c_{5,n}}{c_{3,n}c_{4,n}}\right)+{\left(IR\right)}_{coll,n}+{\left(IR\right)}_{e,n}+{\left(IR\right)}_{mem,n}+\eta_{2,n}-\eta_{1,n}$$ (1)

where E_0_ is the open circuit voltage of VRFBs at 50% state of charge (SOC).

In the stack, the individual cells are connected in series, with the stack voltage expressed as:


$$E_{stack}=\sum_{n}E_{cell}$$ (2)

### 2.1 Mass transfer model

As one of the most important components in vanadium battery, ion exchange membrane plays an important role in isolating vanadium ions from anode and cathode and conducting the internal circuit of vanadium battery. However, ion exchange membrane can not completely prevent vanadium ions from permeating through the membrane. Trace vanadium ions permeate the ion exchange membrane and react with the electrolyte at the other electrode of the battery. The cross-permeation of vanadium ions through the membrane is described by the following reaction equation:


$$V^{2+}+2VO_{2}^{+}+2H^{+}\to 3VO^{2+}+H_{2}O$$ (3)

$$V^{3+}+VO_{2}^{+}\to 2VO^{2+}$$ (4)

$$VO^{2+}+V^{2+}+2H^{+}\to 2V^{3+}+H_{2}O$$ (5)

$$VO_{2}^{+}+2V^{2+}+4H^{+}\to 3V^{3+}+2H_{2}O$$ (6)

Self-reaction caused by vanadium ion cross-permeation introduces the decrease in current efficiency of the battery and the imbalance of active substances in the positive and negative electrolytes, thereby decreasing battery capacity. Therefore, the selective permeability of ion exchange membranes plays a crucial role in determining battery efficiency. The source terms of vanadium ions in porous electrode include two terms, one of which related to electrochemical reaction, and the other related to self-reaction process caused by vanadium ion cross-permeation. The divalent vanadium ion concentration in the porous electrode is calculated by the following equation:


$$\begin{array}{l} \varepsilon V_{e}\frac{\text{d}c_{2,n}}{\text{d}t}=\frac{\omega }{N}\left(c_{2,n}^{res}-c_{2,n}\right)+\frac{V_{e}i_{n}}{d_{e}F}\;-\left(\frac{S}{d_{mem}}D_{2,diff}^{m}-D_{2,E}^{m}I_{n}S\right)c_{2,n}\\ -\left(\frac{S}{d_{mem}}D_{4,diff}^{m}+D_{5,E}^{m}I_{n}S\right)c_{5,n}-2\left(\frac{S}{d_{mem}}D_{5,diff}^{m}+D_{5,E}^{m}I_{n}S\right)c_{5,n} \end{array}$$ (7)

where $c_{i}$ is the vanadium ion concentration of the electrolyte in the porous electrode. $c_{i}^{res}$ is the vanadium ion concentration in the liquid storage tank. *S* is the effective area of the ion exchange membrane. The subscript ‘i’ (i = 2, 3, 4, 5) represents vanadium ions in different valence states. The current density in the battery is denoted as $i_{n}=I_{n}/A_{mem}$, and the concentration of vanadium ions in other valence states is similar. Vanadium ion is also affected by electric field in the process of cross permeation. The mobility of vanadium ion induced by electric field in the membrane is measured experimentally.

Due to the assuming that the electrolyte flowing into the reservoir from the porous electrode is immediately mixed uniformly, the vanadium ion concentration in the electrolyte reservoir is expressed as:


$$V_{res}\frac{\text{d}c_{i}^{res}}{\text{d}t}=-\omega \left(c_{i}^{res}-\bar{c_{i}}\right)$$ (8)

$c_{i}^{res}$ is the concentration of vanadium ions in the electrolyte reservoir, indicating the concentration of electrolyte flowing into the reservoir.

The electrochemical reaction on the surface of graphite fiber in porous electrode results in the concentration difference of vanadium ions between solution in bulk electrolyte and that on the fiber surface. The concentration polarization of electrochemical reactions on graphite fiber surface greatly affects the overpotential of the battery at the start and end of each charge-discharge periodic process. The mass transfer process for each vanadium ion is determined by the following equation:


$$k_{mt}\left(c_{i,n}-c_{i,n}^{sur}\right)=\pm \frac{i_{n}}{Fad_{e}}$$ (9)

where *a* is the specific surface area of the porous electrode. The local mass transfer coefficients were calculated by fitting the relationship experimentally:


$$k_{mt}=1.6\times 10^{-4}{\vec{v}}^{0.4}$$ (10)

where *v* is the electrolyte flow rate in the porous electrode.

Electrochemical reactions in porous electrodes were calculated through the Butler-Volmer equation:


$$-\frac{i_{n}}{Sd_{e}}=Fk_{1r}\exp\left(-\frac{FE_{1}^{0}}{R}\left(\frac{1}{T_{r}}-\frac{1}{T_{e,n}}\right)\right)\sqrt{c_{2,n}^{sur}c_{3,n}^{sur}}\left[\exp(\frac{\eta_{1,n}F}{2RT_{e,n}})-\exp(-\frac{\eta_{1,n}F}{2RT_{e,n}})\right]$$ (11)

$$\frac{i_{n}}{Sd_{e}}=Fk_{2r}\exp\left(\frac{FE_{2}^{0}}{R}\left(\frac{1}{T_{r}}-\frac{1}{T_{e,n}}\right)\right)\sqrt{c_{4,n}^{sur}c_{5,n}^{sur}}\left[\exp(\frac{\eta_{2,n}F}{2RT_{e,n}})-\exp(-\frac{\eta_{2,n}F}{2RT_{e,n}})\right]$$ (12)

The Mass transfer related parameters of cell stack are depicted in Table 1.

**Table 1:** Mass transfer related parameters of cell stack.

Parameters	Values
a	3.5 × 10^4^ m^-1^
*S*	0.078m^-2^
$k_{1r}$	1.75 × 10^-7^ m/s
$k_{2r}$	6.8 × 10^-7^ m/s
$D_{2,diff}^{m}$	8.768 × 10^-12^ m^2^/s
$D_{3,diff}^{m}$	3.222 × 10^-12^ m^2^/s
$D_{4,diff}^{m}$	6.825 × 10^-12^ m^2^/s
$D_{5,diff}^{m}$	5.9 × 10^-12^ m^2^/s
$D_{2,E}^{m}$	5.243 × 10^-11^ m^3^/(s ∙ A)
$D_{3,E}^{m}$	4.945 × 10^-11^ m^3^/(s ∙ A)
$D_{4,E}^{m}$	5.500 × 10^-11^ m^3^/(s ∙ A)
$D_{5,E}^{m}$	3.497 × 10^-11^ m^3^/(s ∙ A)
$\sigma_{coll}$	1000 S/m
$\sigma_{s}$	100 S/m

### 2.2 Heat transfer model

The reaction rates in vanadium battery increase with the growth of temperature. However, vanadium ions are easy to precipitate at high and low temperature, which limits the operating temperature of vanadium batteries. Therefore, reasonable thermal management system is the basis of normal and steady operation of vanadium battery system. In the stack, considering the significant difference of thermal properties between porous electrode and bipolar plate, the heat transfer process in each cell was modeled separately for two regions. One is the region composed of positive and negative porous electrodes and ion exchange membrane, and the other is the region of bipolar plates.

The charge and discharge process of vanadium battery is accompanied by heat generation and heat dissipation, and the heat generated in the battery stack is carried out by convection heat transfer of electrolyte. The temperature of different parts of vanadium battery varies. The parameters related to temperature, such as reaction rate, electrolyte conductivity, vanadium ion diffusion rate in membrane and electrolyte stability, affect the efficiency of vanadium flow battery system. The temperature of electrode and membrane region is calculated by:


$$\begin{array}{l} {\left(\rho C_{p}\right)}_{e}\left(2V_{e}+V_{mem}\right)\frac{\text{d}T_{e,n}}{\text{d}t}=\;\frac{\omega }{N}{\left(\rho C_{p}\right)}_{e}\left(T_{res}^{-}-T_{e,n}\right)+\frac{\omega }{N}{\left(\rho C_{p}\right)}_{e}\left(T_{res}^{+}-T_{e,n}\right)\\ \;+A_{mem}h_{ce}\left(T_{coll,n}-T_{e,n}\right)+A_{mem}h_{ce}\left(T_{coll,n+1}-T_{e,n}\right)+\sum Q_{i} \end{array}$$ (13)

where $T_{e,n}$ is electrode area temperature and $T_{coll,n}$ is bipolar plate temperature. The heat source terms are shown in Table 2.

**Table 2:** Stack heat source term.

Heat source term	Expression
$Q_{ohm}$	$i_{n}^{2}A_{mem}\left(\frac{d_{mem}}{\sigma_{mem}}+\frac{d_{e}}{\varepsilon^{3/2}\sigma_{e}}\right)$
$Q_{activation}$	$i_{n}A_{mem}\left(\eta_{1,n}\text{+}\eta_{2,n}\right)$
$Q_{reaction}$	$\left(\Delta S_{1}+\Delta S_{2}\right)T_{e}i_{n}\frac{A_{mem}}{F}$
$Q_{crossover}$	$\begin{array}{l} \;A_{mem}D_{2}^{mem}\frac{c_{2}}{d_{mem}}\cdot \left(-\Delta H_{2}\right)+A_{mem}D_{3}^{mem}\frac{c_{3}}{d_{m}}\cdot \left(-\Delta H_{3}\right)\\ +A_{mem}D_{4}^{mem}\frac{c_{4}}{d_{mem}}\cdot \left(-\Delta H_{4}\right)+A_{mem}D_{5}^{mem}\frac{c_{5}}{d_{m}}\cdot \left(-\Delta H_{5}\right) \end{array}$

Electrolyte conductivity is calculated by:


$$\begin{array}{l} \sigma_{e}\text{=35}.\text{716+7.699}\times {\text{SOC}}^{-}\;(\text{negative}\;\text{electrolyte})\\ \sigma_{e}\text{=43.763+12.251}\times {\text{SOC}}^{+}\;(\text{positive electrolyte})\; \end{array}$$ (14)

The energy conservation equations for liquid storage tanks and bipolar plates are expressed as follows:


$${\left(\rho C_{p}\right)}_{e}V_{res}\frac{\text{d}T_{res}^{\pm }}{\text{d}t}=-A_{res}h_{res}(T_{res}^{\pm }-T_{air})-\omega {\left(\rho C_{p}\right)}_{e}\left(T_{res}^{\pm }-\bar{T_{e}}\right)$$ (15)

$${\left(\rho C_{p}\right)}_{coll}V_{coll}\frac{dT_{coll,n}}{dt}=-A_{mem}h_{ce}(T_{coll,n}-T_{e,n})-A_{mem}h_{ce}(T_{coll,n}-T_{e,n+1})+i_{n}^{2}A_{mem}\frac{d_{coll}}{\sigma_{coll}}$$ (16)

The energy conservation equation for the current collectors at both ends of the stack is indicated as:


$${\left(\rho C_{p}\right)}_{e}V_{coll}\frac{\text{d}T_{coll}}{\text{d}t}=-A_{mem}h_{ce}(T_{coll}-T_{e})+A_{mem}h_{air}(T_{air}-T_{coll})+i_{a}^{2}A_{mem}\frac{d_{coll}}{\sigma_{coll}}$$ (17)

where $T_{res}^{\pm }$ and $T_{coll,n}$ are the temperature of the reservoir and bipolar plate respectively. $\bar{T_{e}}$ denotes the average stack outlet temperature.

Parameters related to stack heat transfer are shown in Table 3.

**Table 3:** Parameters related to stack heat transfer.

Parameter	Value
${\left(\rho C_{p}\right)}_{e}$	4.3 × 10^6^ J/(m·K)
${\left(\rho C_{p}\right)}_{c}$	4.03 × 10^6^ J/(m·K)
$-\Delta S_{1}$	−100 J/(mol·K)
$-\Delta S_{2}$	−21.7 J/(mol·K)
$\Delta H_{2}$	−2.2 × 10^5^ J/mol
$\Delta H_{3}$	−6.4 × 10^4^ J/mol
$\Delta H_{4}$	−9.12 × 10^4^ J/mol
$\Delta H_{5}$	−2.468 × 10^5^ J/mol
$\lambda_{e}$	0.67 W/(m·K)
$\lambda_{air}$	0.0257 W/(m·K)

### 2.3 Shunt current model

**Fig 2:**
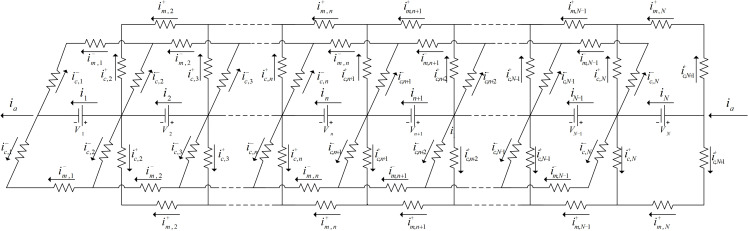
Battery stack circuit model.

Because that the cells in the stack are connected in series, the potential between different cells varies. Electrolyte, allocated from the manifold and flow channel in the battery stack, flows through different single cells. Thus, it generates shunt current resulted from the potential difference between cells, which reduces the current efficiency. The circuit model of battery stack is shown in Fig 2.

The currents in the manifold, flow channels and each cell are calculated by the Kirchhoff's current law:


$$I_{m,n+1}^{\text{+}}-I_{m,n}^{\text{+}}-I_{c,n+1}^{\text{+}}=0$$ (18)

$$I_{m,n+1}^{\text{-}}-I_{m,n}^{\text{-}}-I_{c,n+1}^{\text{-}}=0$$ (19)

$$I_{n+1}-I_{n}+2I_{c,n+1}^{+}+2I_{c,n+1}^{\text{-}}=0$$ (20)

$$\text{-}r_{m}^{\text{-}}I_{m,n}^{\text{-}}+r_{c}^{\text{-}}\left(I_{c,n+1}^{\text{-}}-I_{c,n}^{\text{-}}\right)\text{+}r_{e}I_{n}=E_{n}$$ (21)

$$\text{-}r_{m}^{\text{+}}I_{m,n}^{+}+r_{c}^{+}\left(I_{c,n+1}^{\text{+}}-I_{c,n}^{\text{+}}\right)\text{+}r_{e}I_{n}=E_{n}$$ (22)

where *r* is the internal resistance of the single cell and *I* is the current in cells. Subscripts m, e and c denote the membranes, electrodes and collectors respectively. Superscript +  and − indicate positive and negative electrodes of the battery. The equations for a single cell at both ends of the stack are $I_{m,1}^{-}=-I_{c,1}^{-}$, $I_{m,2}^{\text{+}}=-I_{c,2}^{+}$, $I_{m,N\text{-}1}^{-}=I_{c,N}^{-}$ and $I_{m,N}^{+}=I_{c,N+1}^{+}$.

## 3. Results and discussion

### 3.1 Model validation

**Fig 3:**
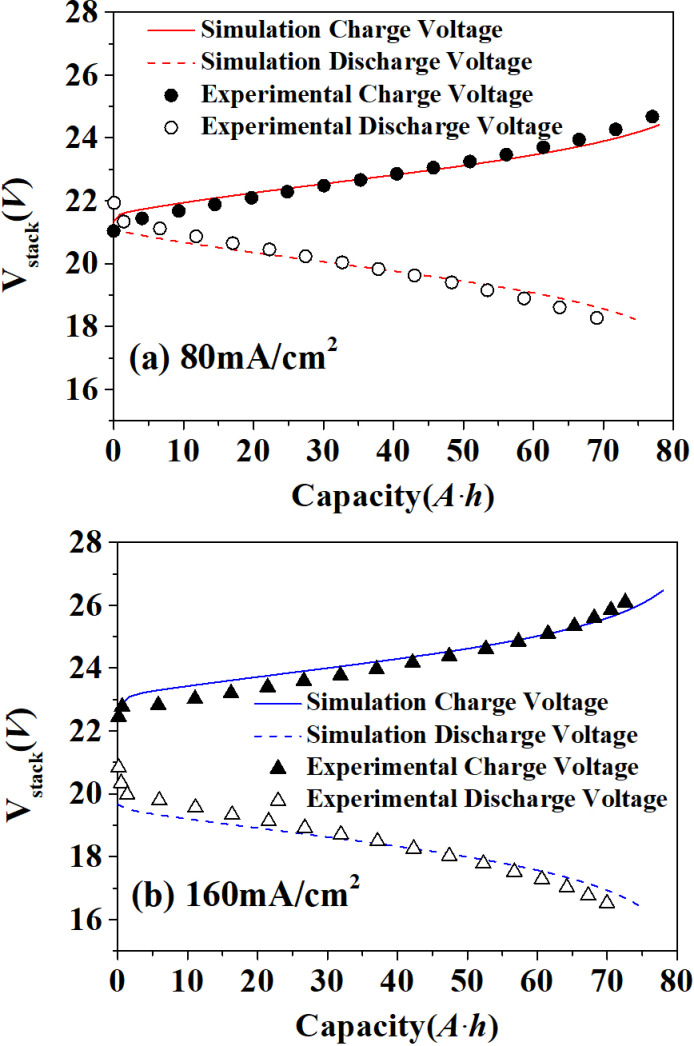
Comparison between simulation and experimental results of charge-discharge voltage.

The calculated parameters of vanadium battery model in this paper are consistent with the experimental parameters in literature [24]. The stack consisted of 15 single cells, and each single cell is stacked in series to form a kilowatt stack module. Nafion 115 ion exchange membrane was used as vanadium battery separator. Its effective area was 780 cm^-2^ and the thickness of membrane was 0.127 mm. The charge state during the charging and discharging process is controlled between 15% and 85%. The mass and heat transfer equations were solved by fourth-order Runge-Kutta method with a time step of 1s. As shown in Fig 3, there is little difference between the simulated voltage and the experimental voltage during the charging and discharging process, indicating the significant accuracy of the developed model. Voltage efficiency at different temperature are shown as data in S1 Fig.

### 3.2 Analysis of results

For large-scale flow battery system, reasonable operation parameters are an important guarantee for high efficiency and stable operation of the system. According to the calculation results of non-isothermal model established, the influence of vanadium ion total concentration change and current density and electrolyte temperature associated with imbalance of vanadium ion concentration in vanadium flow battery stack during charge and discharge are analyzed.

#### 3.2.1 Analysis of vanadium ion imbalance.

**Fig 4:**
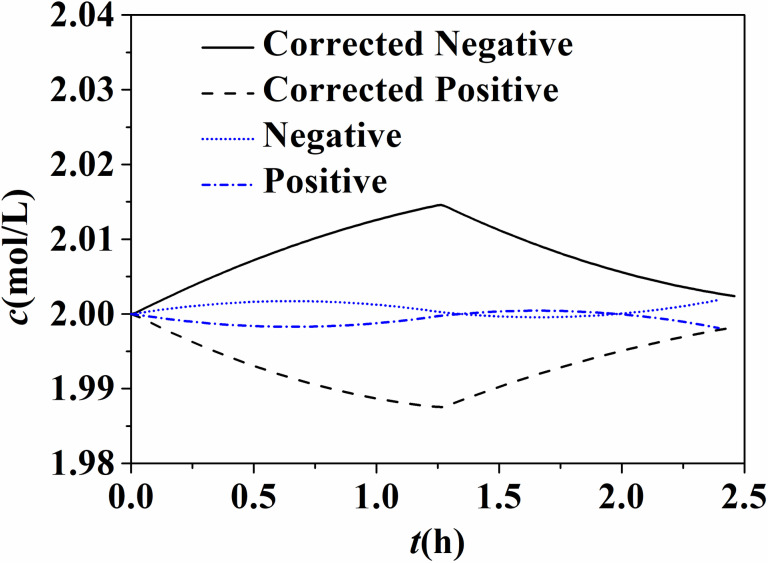
Total vanadium ion concentration in liquid storage tank (80 mA/cm^2^, 298 K).

In this paper, the permeating process of vanadium ions in ion exchange membrane is described completely, including both diffusion of vanadium ions due to concentration gradient and electric field effect in ion exchange membrane. Fig 4 shows the variation curve of the total concentration of vanadium ions in the positive and negative electrodes of the storage tank, at temperature of 298K and current density of 80 mA/cm^2^. It also shows that only diffusion of vanadium ions in different valence states is considered in non-corrected model. The total vanadium ion concentration in cathode is slightly higher than that in anode after the whole charge-discharge cycle, which indicates that vanadium ion permeates from anode to cathode. After the correction of the flow battery model, the permeation of positively charged vanadium ions in the ion exchange membrane is affected by the electric field, which promotes the permeation of tetravalent and pentavalent vanadium ions to the negative electrode and inhibits the permeation of divalent and trivalent vanadium ions to the positive electrode during charging stage. During discharging stage, it promotes the permeation of divalent and trivalent vanadium ions to the positive electrode, while inhibiting the migration of tetravalent and pentavalent vanadium ions to the negative electrode. Therefore, the total vanadium ion concentration of negative electrode increases during charging and decreases during discharging, and that of positive electrode is opposite. The total vanadium ion concentration of cathode is higher than that of anode at the end of discharge stage.

#### 3.2.2 Effect of operating parameters on vanadium ion concentration in stack.

In the process of charge and discharge, reasonable current density, electrolyte temperature and other operating parameters are the premise to ensure the efficient and stable operation of vanadium battery. For the VRFB stack composed of multiple cells connected in series, the current in the circuit of different cells is different due to the conduction of electrolyte in the flow channel, and vanadium ions permeation in the ion exchange membrane of each cell. Thus, it causes the concentration of vanadium ions in various valence states in the electrolyte to be different. The electrolyte temperature of vanadium battery affects the mass transfer, electrochemical reaction rate and equilibrium potential of vanadium ion in the stack significantly. Average temperature at stack outlets (SOC = 50%) is shown as data in S2 Fig.

**Fig 5:**
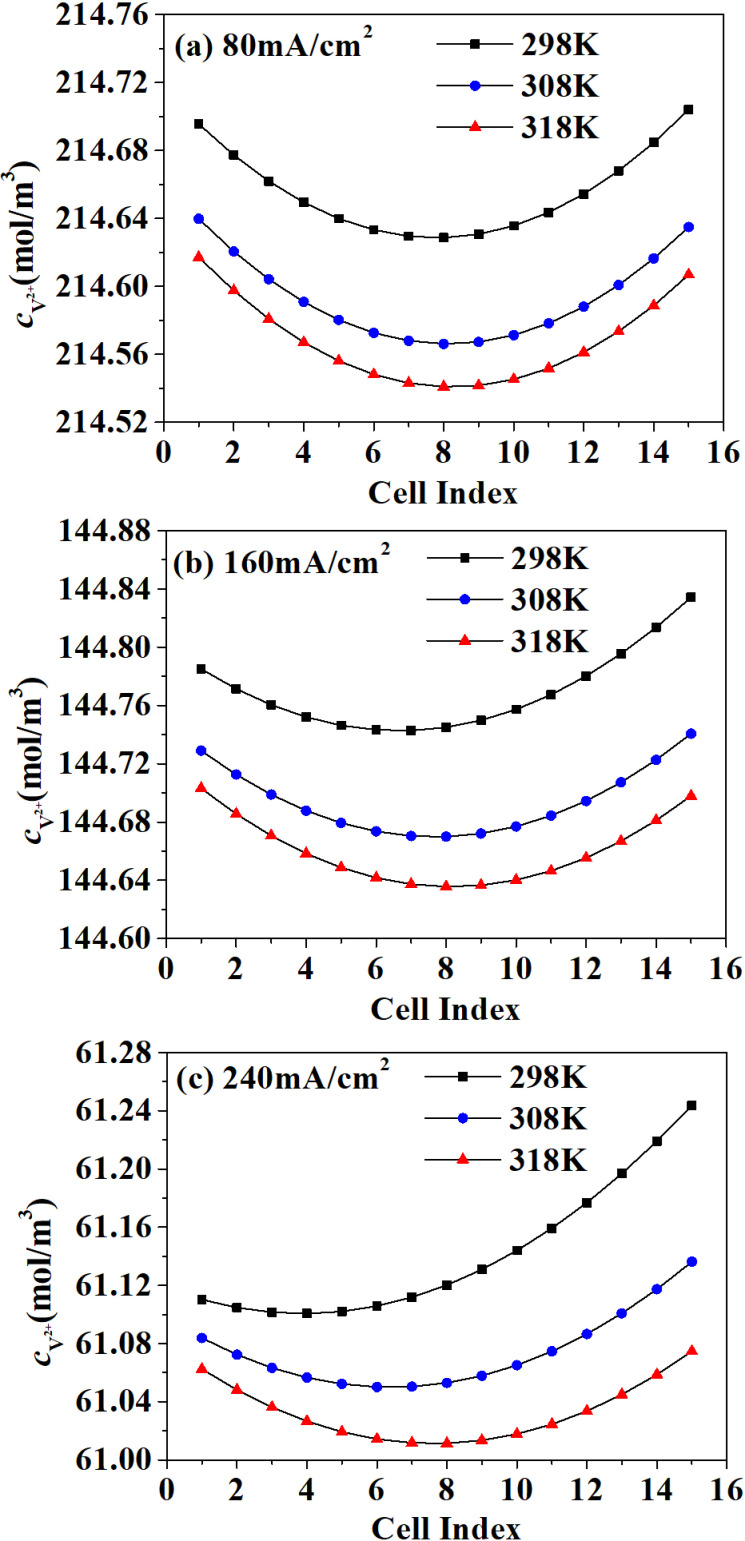
V^2 +^ concentration.

Taking divalent vanadium ion (V^2+^) as an example, as shown in Fig 5, the results indicate the vanadium ion concentration in each single cell at the end of discharge. It can be seen from the results that the vanadium ion concentration in the single cell near the middle of the stack is lower, indicating that the electrochemical reaction current in the middle single cell is smaller than the other cells in stack. At lower electrolyte temperature, the concentration of vanadium ions in the membrane increased with the increase of current density, indicating that the permeation of vanadium ions in the membrane increased sharply with the growth of current density. At the same current density, vanadium ion concentration tends to be more uniform when electrolyte temperature increases.

## 4. Conclusions

A mathematical model including equivalent circuit, heat and mass transfer and electrochemical reaction of VRFB system is established. The charge-discharge voltage obtained agrees well with the experimental results. The imbalance of vanadium ion concentration in the storage tank of vanadium flow battery is investigated. Moreover, the influence of battery operating parameters on the imbalance of vanadium ion concentration in the electrolyte among each cell of battery stack is studied. The results show that the permeation process of vanadium ions in ion exchange membrane is greatly affected by electric field, and the total concentration of vanadium ions in negative electrode is higher than that in positive electrode at the end of charge and discharge. At low temperature, the current density has a great influence on vanadium ion imbalance in each cell. While at high electrolyte temperature, the influence of current density decreases and can be almost ignored. This model provides a good basis for the study of operating parameters required for efficient and long-term stable operation of large-scale VRFB system.

## Supporting Information

S1 Fig Voltage efficiency at different temperature. (DOCX)

S2 Fig Average temperature a at stack outlets (SOC = 50%). (DOCX)
