# Supplementary material for: Simulation of the electrolyte imbalance in vanadium redox flow batteries
Source: PLoS One. 2025 Feb 7;20(2):e0318460. doi: 10.1371/journal.pone.0318460 (PMC11805378; doi:10.1371/journal.pone.0318460)
Supplement: S2 Fig — (DOCX) [file pone.0318460.s002.docx]

***Simulation of the electrolyte imbalance in vanadium redox flow batteries***

**Baowen Zhang^1,2*^, Yuan Lei^3^**

^1^ Aviation Engineering School, Air Force Engineering University, Xi'an, P. R. China

^2^ Equipment Management and Unmanned Aerial Vehicle Engineering School, Air Force Engineering University, Xi'an, P. R. China

^3^ School of Chemical Engineering, Northwest University, Xi'an, China

* Corresponding author: bwzhangr@foxmail.com (BZ)


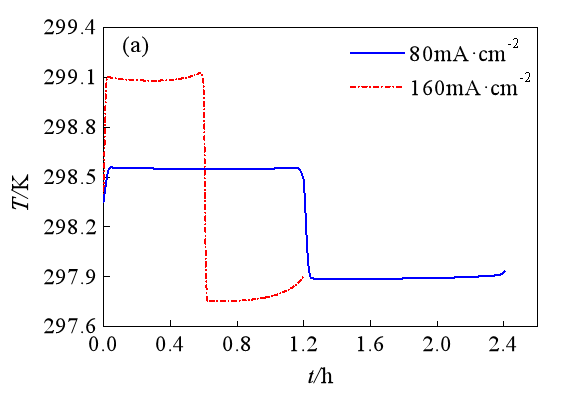


S2 Fig. Average temperature a at stack outlets (SOC=50%).
